# Validation to Brazilian Portuguese of the coma recovery scale-revised

**DOI:** 10.1055/s-0044-1791657

**Published:** 2024-11-20

**Authors:** Ana Paula Silva Champs, Thayse Nayara Freitas do Vale Santanna, Christian Marques Couto, Roberta Correa Macedo, Patricia Sola Penna, Luciana Charchar Vilas Boas Cruz, Rafael Xavier da Silva-Neto, Luiz Sérgio Vaz

**Affiliations:** 1 Rede SARAH de Hospitais de Reabilitação, Belo Horizonte MG, Brazil.; 2 Rede SARAH de Hospitais de Reabilitação, Salvador BA, Brazil.; 3 Rede SARAH de Hospitais de Reabilitação, Rio de Janeiro RJ, Brazil.

**Keywords:** Brain Injuries, Consciousness Disorders, Rehabilitation, Validation Study, Lesões Encefálicas, Transtornos da Consciência, Reabilitação, Estudo de Validação

## Abstract

**Background** To improve the diagnostic accuracy of the state of consciousness of patients with severe brain injury, Giacino et al. introduced the Coma Recovery Scale (CRS) in 1991, which underwent revision in 2004, resulting in the revised CRS scale (CRS-R).

**Objective** To determine the concurrent validity, as well as inter- and intrarater agreement of the CRS-R's adaptation to Brazilian Portuguese.

**Methods** This study involved a sample of 30 patients with severe brain injury. Concurrent evaluations were also performed with the Glasgow Coma Scale (GCS) and the Full Outline of UnResponsiveness (FOUR) scale. A total of seven rehabilitation experts were recruited to assess the inter- and intrarater reliability agreement.

**Results** Interrater reliability was moderate to high for auditory, visual, motor, verbal, communication, and arousal subscales (Cohen weighted kappa = 0.765 to 0.892; *p* < 0.001). Significant inter and intrarater intraclass correlation coefficients were observed for the total CRS-R scores, all of which were statistically significant ( *p* < 0.001). Also, total CRS-R scores exhibited a high correlation with the total GCS and FOUR scores, indicating acceptable concurrent validity ( *p* < 0.001).

**Conclusion** The Brazilian Portuguese version of CRS-R can be reliably administered by trained examiners. This study demonstrated substantial to almost perfect interrater agreement for the total score and subscales, as well as high concurrent validity between the Brazilian Portuguese version of CRS-R and the other two standardized behavioral scales.

## Resumo

**Antecedentes** Para melhorar a acurácia diagnóstica do estado da consciência de pacientes com lesão cerebral severa, foi desenvolvida a Escala de Recuperação do Coma em 1991, que passou por revisão em 2004, resultando na escala revisada (CRS-R).

**Objetivo** Determinar a validade concorrente, concordância inter- e intraexaminador da adaptação da CRS-R para o português do Brasil.

**Métodos** Este estudo envolveu uma amostra de 30 pacientes com lesão cerebral grave. As avaliações concorrentes foram realizadas com a Escala de Coma de Glasgow (ECG) e a escala Full Outline of UnResponsiveness (FOUR). Um total de sete especialistas em reabilitação foram recrutados para avaliar a concordância de confiabilidade inter- e intraexaminador.

**Resultados** A confiabilidade interexaminador foi de moderada a alta para as subescalas auditiva, visual, motora, verbal, comunicação e alerta (kappa ponderado de Cohen = 0,765 a 0,892; *p* < 0,001). Foram observados coeficientes de correlação intraclasse intra- e interexaminador significativos para os escores totais da CRS-R, todos estatisticamente significativos ( *p* < 0,001). Adicionalmente, os escores totais da CRS-R apresentaram alta correlação com os escores ECG e FOUR totais, indicando validade concorrente aceitável ( *p* < 0,001).

**Conclusão** A versão em português do Brasil da CRS-R pode ser administrada de forma confiável por examinadores treinados. Este estudo demonstrou uma concordância de confiabilidade interexaminador substancial a quase perfeita para os escores total e de subescalas, assim como alta validade concorrente entre a versão brasileira do CRS-R e as outras duas escalas comportamentais.

## INTRODUCTION

Severe brain injuries from trauma, infections/inflammation, or vascular events significantly impact patients and their families, resulting in the need for complex care and prompting transformative changes in their lives. With improved access to care resources like emergency services and rehabilitation departments, the survival rates of patients bearing sequelae of such injuries have increased. One of the foremost clinical challenges for these patients is evaluating consciousness, especially in those who appear nonresponsive. 1

Although most patients with brain injuries emerge from coma within days, some can progress to a complete loss of brain functions. Furthermore, others may display varying degrees of interaction with their surroundings or undergo different recovery phases before achieving complete or partial restoration of consciousness. 2 3 4 5

Disorders of Consciousness (DoC) are typically described as extended states of altered consciousness, evident after 28 days postbrain injury. 6 7 The spectrum includes conditions ranging from coma to full consciousness. For instance, individuals described as being in a vegetative state (VS) or unresponsive wakefulness syndrome (UWS) are conscious but unaware. 6 7 8 Minimally conscious state (MCS) individuals exhibit limited but consistent awareness of themselves or their environment. Those in the minimally conscious state minus (MCS-) only show voluntary responses to nociceptive stimuli and can visually fixate on objects without localization. 9 10 Conversely, minimally conscious state plus (MCS + ) patients can follow commands, produce intelligible speech, and establish purposeful communication. 11 Emergence from MCS (eMCS) signifies a functional use of objects and communication abilities. 6 7 The locked-in syndrome is characterized by awareness of one's surroundings, sustained eye-opening, and specific eye movements as a communication means. 11 12 13 In rarer cases, a dissociated cognitive state, for example, the functional locked-in syndrome, manifests when patients do not seem to follow commands yet demonstrate brain activity in complementary exams, such as somatosensory potential and video-electroencephalogram. 11 12 13

Preserving a patient's autonomy is paramount. However, envision a scenario where an individual cannot eat, drink, speak, or communicate. If even their families are uncertain about their state of consciousness, the situation becomes profoundly distressing. This underscores the importance of detecting consciousness in noncommunicative patients with severe brain injuries. Misdiagnoses can be catastrophic. To enhance diagnostic accuracy, several scales have been developed. Notably, Giacino et al. introduced the coma recovery scale (CRS) in 1991, which underwent revision in 2004, resulting in a revised scale (CRS-R). 14 15

The CRS-R is a comprehensive tool that evaluates various consciousness states, aiding in both diagnosis and prognosis. Standardizing data fosters consistent information sharing and comparative studies across nations. The scale evaluates auditory, visual, verbal, and motor functions, communication, and arousal levels. Its total score can range from 0 (worst) to 23 (best). 15

This research endeavors to translate and validate the 2004 version of CRS-R for the Brazilian Portuguese language.

## METHODS

### Translation and back translation

The original scale's creators authorized its translation from English to Brazilian Portuguese. The initial version was done by three independent bilingual translators. Subsequently, they collaborated to formulate a consensus version of the scale, which was then back-translated by two additional bilingual translators. Upon completing the backwards translation, both the original authors and the research group reviewed it for accuracy, culminating in the development of the CRS-R or, in Portuguese, Escala de Recuperação do Coma-Revisada (ERC-R) ( Supplementary Material S1 - https://www.arquivosdeneuropsiquiatria.org/wp-content/uploads/2024/07/ANP-2023.0315-Supplementary-Material.docx , online only).

### Participants

Individuals were recruited from a Brazilian network of rehabilitation hospitals during their rehabilitation programs. Inclusion criteria were a diagnosis of brain injury and a suspected altered state of consciousness. Those with neurological disorders unrelated to brain injury were excluded. A total of 30 consecutive patients with severe brain injuries were included in the study. Clinical and epidemiological data were extracted from medical records. Legal guardians of all participants provided written consent, following approval from the ethical review board of the Network of Rehabilitation Hospitals (CAAE 35466920.7.1001.0022) in Belo Horizonte, Brazil.

### Procedures

There were seven professionals from three units of a Brazilian Network of Rehabilitation Hospitals centers who participated to assess interrater agreements, with three from Belo Horizonte, two from, and two from Rio de Janeiro. The group consisted of internal medicine physicians ( *n* = 3), a physiotherapist ( *n* = 1), and neurologists ( *n* = 3). These professionals underwent training in using the CRS-R scale, utilizing a video ( Supplementary Material S2 - https://www.sralab.org/sites/default/files/downloads/2020-09/Training_Modules_20200831_v1.pdf ) that demonstrated the administration procedures for each item with real patients (online only).

Following training, the professionals' assessment protocol was:

- Day 1: Rater A and Rater B independently administered the Brazilian-Portuguese version of the scale (∼1 hour apart) to evaluate inter-rater reliability.
- Day 2: Rater A (or Rater B) re-administered the Brazilian-Portuguese version to assess test-retest reliability.

Raters A and B were blinded to each other's evaluations during this process.

The CRS-R total scores were compared with total scores from the Glasgow coma scale (GCS) and full outline of unresponsiveness (FOUR) scale to determine concurrent validity.

### Data analysis

Data analysis was conducted using the R software (R Foundation for Statistical Computing, Vienna, Austria), version 4.1.2, with a significance level of 0.05. Descriptive statistics were employed to describe the clinical and epidemiological characteristics of the participants. Interrater reliability was assessed using the Cohen kappa tests. 16 This test measured the reproducibility of CRS-R's total and subscale scores across different raters. The agreement coefficients resulting from the kappa tests were categorized as follows: poor < 0; slight = 0.01 to 0.19; fair = 0.20 to 0.39; moderate = 0.40 to 0.59; substantial = 0.60 to 0.79; and almost perfect = 0.80 to 1.00. 16 For concurrent validity, scores from the three scales (CRS-R, GCS, and FOUR) were analyzed using Spearman correlation. The alpha level was 0.05.

## RESULTS

A total of 30 patients with severe brain injury, suspected of having altered states of consciousness, were evaluated. Of these patients, 70% were male, with an average age of 29.5 ± 9.8 years. In terms of states of consciousness, 34.4% patients were diagnosed with a VS/UWS, 41.3% as MCS -, 6.8% as MCS +, and five patients had emerged from MCS. On average, the duration since brain injury onset, or ictus, was 12 months.

Trauma was the cohort's leading cause of brain injury, accounting for 70%, followed by anoxia with 23.3% of cases. Surgical intervention, either in the form of a craniotomy or craniectomy, was undergone by 23.3% of the patients. Neurologically, 89.5% of the patients exhibited reactive pupils, 98.6% had a corneal reflex, 68.1% displayed skew or conjugate deviation, 20.6% showed abnormal flexion, and 96.4% presented spasticity. Other accompanying symptoms included constipation (50%), seizures (30%), pain (26.6%), sleep disturbances (53%), and sialorrhea (12.5%). Further details can be found in Table 1 .

**Table 1:** Clinical and epidemiological profile ( *n* = 30)

Characteristics		N ^o^ . of cases (%)/ mean or median
**Gender**	Female	9 (30%)
	Male	21 (70%)
**Age**	Mean (SD)	29.5 (± 9.8)
Time from brain injury ^†^	Median (Q _1_ –Q _3_ )	7.5 (5.0–17.5)
**Etiology**	Traumatic	21 (70%)
	Anoxia	7 (23.3%)
	Stroke	1 (3.3%)
	Other	1 (3.3%)
**Brain surgery**	Yes	7 (23.3%)
	No	23 (76.7%)
** CRS–R classification ^‡^**	VS/UWS	11 (36.6%)
	MCS-	8 (26.6%)
	MCS+	5 (16.6%)
	eMCS	6 (20%)
**Neurological and clinical findings**	Pupils' reaction and symmetry	28 (89.5%)
	Skew or conjugate deviation	13 (43.3%)
	Abnormal flexion	6 (20%)
	Spasticity	29 (96.6%)
	Constipation	8 (26.6%)
	Seizures	9 (30%)
	Sleep disturbances	8 (26.6%)
	Sialorrhea	4 (13.3%)

**Abbreviations:** CRS-R, Coma Recovered Scale Revised; eMCS, emergence from minimally conscious state; FOUR, Full Outline of UnResponsiveness; MCS-, minimally conscious state minus; MCS + , Minimally Conscious State Plus; SD, standard deviation; VS/UWS, vegetative state/unresponsive wakefulness syndrome. **Notes:** ^†^ In months. ^**‡**^ Day 1, rater A.

Intrarater reliability for CRS-R auditory, visual, motor, oromotor/verbal, communication, and arousal subscales ranged from strong to almost perfect (weighted kappa: 0.828–0.993; *p* < 0.001). The total score also demonstrated significant reliability with a kappa value of 0.97. In the interrater analysis, the subscales showed moderate to strong (weighted kappa: 0.69–0.87; *p* < 0.001), with the total score having a kappa value of 0.96. Considering all the collected data, the total CRS-R scores exhibited a high correlation (0.50–0.70) with total GCS (Spearman rho = 0.680; *p* < 0.001) and total FOUR (Spearman rho = 0.644; *p* < 0.001), indicating an acceptable concurrent validity.

Detailed results are presented in Tables 2 , 3 , 4 , and 5 .

**Table 2:** Intrarater analysis - agreement proportion between 2 days and weighted Kappa Intra-rater reliability – rater A

Variable		Agreement (%)	Squared weighted Kappa	95% CI	*p* -value	Intra-rater reliability
**Glasgow scale**	Eye opening	90	0.348	-0.087; 0.999	0.051	Minimal
	Verbal response ^†^	82.8	0.732	0.310; 0.965	< 0.001	Moderate
	Motor response	93.3	0.949	0.828; 0.999	< 0.001	Almost perfect
**FOUR scale**	Eye response	86.7	0.737	0.494; 0.933	< 0.001	Moderate
	Motor response	90	0.916	0.752; 0.999	< 0.001	Almost perfect
	Brainstem reflexes	100	1.000	no variance	< 0.001	Almost perfect
**CRS-R**	Auditory function	83.3	0.950	0.803; 0.984	< 0.001	Almost perfect
	Visual function	83.3	0.959	0.854; 0.992	< 0.001	Almost perfect
	Motor function	93.3	0.993	0.974; 0.999	< 0.001	Almost perfect
	Oromotor/verbal function ^†^	72.4	0.847	0.680; 0.936	< 0.001	Strong
	Communication	90	0.919	0.824; 0.999	< 0.001	Almost perfect
	Arousal	86.7	0.828	0.465; 0.977	< 0.001	Strong
			** ICC (3.1) ^‡^**			
Glasgow total		73.3	0.912	0.823; 0.957	< 0.001	Excellent
FOUR total ^†^		89.3	0.963	0.922; 0.983	< 0.001	Excellent
CRS-R total		60.0	0.975	0.948; 0.988	< 0.001	Excellent

Abbreviations: CI, confidence interval; CRS-R, Coma Recovered Scale Revised; FOUR, Full Outline of UnResponsiveness; ICC, intraclass correlation.

**Notes:**^†^ n = 29 pairs. ^**‡**^ ICC (3.1), two-way mixed models, single measurement, absolute agreement. For the assessment of concurrent validity, scores from the three scales (CRS-R, GCS, and FOUR) were analyzed using Intraclass Correlation Coefficients (ICC) (two-way mixed models, single measurement and absolute agreement) for inter-rater and intra-rater reliability.

**Table 3:** Intra-rater analysis - agreement -roportion between 2 days and weighted Kappa intrarater reliability – rater B

Variable		Agreement (%)	Squared weighted Kappa	95% CI	*p* -value	Intrarater reliability
**Glasgow scale**	Eye opening	90	0.545	0.000; 0.795	0.002	Weak
	Verbal response†	89.7	0.830	0.408; 0.999	< 0.001	Strong
	Motor response	93.3	0.975	0.926; 0.999	< 0.001	Almost perfect
**FOUR scale**	Eye response	93.3	0.717	0.365; 0.999	< 0.001	Moderate
	Motor response	93.3	0.966	0.899; 0.999	< 0.001	Almost perfect
	Brainstem reflexes	100	1.000	no variance	< 0.001	Almost perfect
**CRS-R**	Auditory function	80	0.948	0.889; 0.983	< 0.001	Almost perfect
	Visual function	86.7	0.951	0.865; 0.994	< 0.001	Almost perfect
	Motor function	80	0.962	0.897; 0.990	< 0.001	Almost perfect
	Oromotor/verbal function ^†^	75.9	0.837	0.669; 0.931	< 0.001	Strong
	Communication	93.3	0.946	0.840; 0.999	< 0.001	Almost perfect
	Arousal	83.3	0.811	0.485; 0.964	< 0.001	Strong
			** ICC (3.1) ^‡^**			
Glasgow total		76.7	0.931	0.856; 0.967	< 0.001	Excellent
FOUR total ^†^		86.7	0.961	0.919; 0.981	< 0.001	Excellent
CRS-R total		60.0	0.971	0.938; 0.986	< 0.001	Excellent

Abbreviations: CI, confidence interval; CRS-R, Coma Recovered Scale Revised; FOUR, Full Outline of UnResponsiveness; ICC, intraclass correlation.

Notes: ^†^ n = 29 pairs. ^**‡**^ ICC (3.1), two-way mixed models, single measurement, absolute agreement. For the assessment of concurrent validity, scores from the three scales (CRS-R, GCS, and FOUR) were analyzed using ICC - Intraclass Correlation Coefficients (two-way mixed models, single measurement and absolute agreement) for inter-rater and intra-rater reliability.

**Table 4:** Inter-rater analysis - agreement proportion between 2 raters and weighted Kappa inter-rater reliability - day 1

Variable		Agreement (%)	Weighted Kappa	95% CI	*p* -value	Inter-rater reliability
**Glasgow scale**	Eye opening	86.7	0.259	-0.262; 0.781	0.330	Minimal
	Verbal response ^†^	72.4	0.689	0.503; 0.874	< 0.001	Moderate
	Motor response	93.3	0.917	0.803; 0.999	< 0.001	Almost perfect
**FOUR scale**	Eye response	73.3	0.689	0.503; 0.874	< 0.001	Moderate
	Motor response	76.7	0.762	0.593; 0.931	< 0.001	Moderate
	Brainstem reflexes	90	0.483	0.056; 0.909	0.026	Weak
**CRS-R**	Auditory function	80	0.819	0.676; 0.963	< 0.001	Strong
	Visual function	66.7	0.791	0.670; 0.912	< 0.001	Strong
	Motor function	76.7	0.872	0.772; 0.971	< 0.001	Strong
	Oromotor/verbal function ^†^	72.4	0.696	0.501; 0.890	< 0.001	Moderate
	Communication	86.7	0.820	0.657; 0.983	< 0.001	Strong
	Arousal	83.3	0.761	0.547; 0.974	< 0.001	Moderate
			** ICC (3.1) ^‡^**			
Glasgow total		56.7	0.920	0.839; 0.961	< 0.001	Excellent
FOUR total ^†^		56.7	0.865	0.735; 0.933	< 0.001	Good
CRS-R total		53.3	0.961	0.920; 0.981	< 0.001	Excellent

Abbreviations: CI, confidence interval; CRS-R, Coma Recovered Scale Revised; FOUR, Full Outline of UnResponsiveness; ICC, intraclass correlation.

Notes: ^†^ n = 29 pairs. ^**‡**^ ICC (3.1), two-way mixed models, single measurement, absolute agreement. For the assessment of concurrent validity, scores from the three scales (CRS-R, GCS, and FOUR) were analyzed using ICC - Intraclass Correlation Coefficients (two-way mixed models, single measurement and absolute agreement) for inter-rater and intra-rater reliability.

**Table 5:** Inter-rater analysis - agreement proportion between 2 raters and weighted Kappa Inter-rater reliability - day 2

Variable		Agreement (%)	Weighted Kappa	95% CI	*p* -value	Inter-rater reliability
**Glasgow scale**	Eye opening	93.3	0.483	0.136; 0.830	0.006	Weak
	Verbal response ^†^	75.9	0.728	0.537; 0.919	< 0.001	Moderate
	Motor response	93.3	0.934	0.846; 0.999	< 0.001	Almost perfect
**FOUR scale**	Eye response	76.7	0.533	0.255; 0.812	< 0.001	Weak
	Motor response	86.7	0.881	0.765; 0.997	< 0.001	Strong
	Brainstem reflexes	89.7	0.480	0.053; 0.907	0.028	Weak
**CRS-R**	Auditory function	70.0	0.755	0.608; 0.901	< 0.001	Moderate
	Visual function	80.0	0.853	0.740; 0.965	< 0.001	Strong
	Motor function	73.3	0.825	0.693; 0.958	< 0.001	Strong
	Oromotor/verbal function ^†^	82.8	0.826	0.680; 0.972	< 0.001	Strong
	Communication	96.7	0.958	0.876; 0.999	< 0.001	Almost perfect
	Arousal	86.7	0.798	0.591; 0.999	< 0.001	Strong
			** ICC (3.1) ^‡^**			
Glasgow total		73.3	0.958	0.914; 0.980	< 0.001	Excellent
FOUR total ^†^		69.0	0.874	0.748; 0.939	< 0.001	Good
CRS-R total		60.0	0.963	0.924; 0.982	< 0.001	Excellent

Abbreviations: CI, confidence interval; CRS-R, Coma Recovered Scale Revised; FOUR, Full Outline of UnResponsiveness; ICC, intraclass correlation.

Notes: ^†^ n = 29 pairs. ^**‡**^ ICC (3.1), two-way mixed models, single measurement, absolute agreement. For the assessment of concurrent validity, scores from the three scales (CRS-R, GCS, and FOUR) were analyzed using ICC - Intraclass Correlation Coefficients (two-way mixed models, single measurement and absolute agreement) for inter-rater and intra-rater reliability.

Table 6 illustrates the detection of signs of consciousness using CRS-R compared with GCS and FOUR for 30 patients. Visual pursuit and/or fixation were not detected in seven cases (23.3%) with GCS. Meanwhile, the FOUR did not detect visual fixation in two patients (6.7%).

**Table 6:** The detection of signs of consciousness across CRS-R and GCS, FOUR ( *n* = 30) ^†^

Participant	CRS-R	GCS	FOUR
1	VS/UWS	No signs	No signs
2	VS/UWS	No signs	No signs
3	VS/UWS	No signs	No signs
4	eMCS	Full consciousness	Full consciousness
5	VS/UWS	No signs	No signs
6	MCS- (visual pursuit)	No signs	Eyelids opening and tracking
7	VS/UWS	No signs	No signs
8	VS/UWS	No signs	No signs
9	MCS- (visual fixation)	No signs	No signs
10	VS/UWS	No signs	No signs
11	VS/UWS	No signs	No signs
12	MCS+ (consistent movement to command)	Obey command	Makes sign
13	eMCS	Full consciousness	Full consciousness
14	MCS+ (consistent movement to command)	Obey command	Makes sign
15	MCS- (visual pursuit)	No signs	Eyelids opening and tracking
16	MCS- (visual pursuit)	No signs	Eyelids opening and tracking
17	VS/UWS	No signs	No signs
18	eMCS	full consciousness	Full consciousness
19	VS/UWS	No signs	No signs
20	MCS- (localization to noxious stimulation)	Moves to localized pain	Localizing to pain
21	eMCS	Full consciousness	Full consciousness
22	eMCS	Full consciousness	Full consciousness
23	MCS- (visual fixation)	No signs	No signs
24	MCS- (visual fixation)	No signs	No signs
25	MCS+ (consistent movement to command)	Obey command	Localizing to pain
26	MCS- (visual pursuit)	No signs	Eyelids opening and tracking
27	MCS+ (consistent movement to command)	Obey command	Eyelids opening and tracking)
28	VS/UWS	No signs	No signs
29	eMCS	Signs of consciousness	Signs of consciousness
30	MCS+ (consistent movement to command)	Obey command	Localizing to pain

Abbreviations: CRS-R, Coma Recovery Scale Revised; eMCS, emergence from minimally conscious state; FOUR, FOUR, Full Outline of UnResponsiveness; GCM, Glasgow Coma Scale; MCS-, minimally conscious state minus; MCS + , minimally conscious state plus; VS/UWS, vegetative state/unresponsive wakefulness syndrome.

Notes: ^†^ Day 1, rater A.

## DISCUSSION

Assessing patients with brain injuries and suspected DoC is challenging. Utilizing a structured clinical assessment is crucial to avoid misdiagnoses. 6 7 The findings from this study suggest that the CRS-R's Brazilian Portuguese translation provides reproducible results across different examiners. There was substantial to almost perfect interrater agreement observed for the total score and subscales. Furthermore, high concurrent validity was established between the Brazilian Portuguese Version of CRS-R and two other standardized behavioral scales, namely GCS and FOUR. 17 18

The interrater reliability, as denoted by the correlation coefficient between total scores (K = 0.96), was similar to what has been observed in other validation studies with Latin languages: European Portuguese at 0.87, 19 French at 0.80, 20 Italian at 0.81, 21 and Spanish at 0.97. 22

The CRS-R demonstrated superior detection of signs of consciousness compared with GCS and FOUR. Specifically, visual pursuit and/or fixation were no detected in seven cases (23.3%) using GCS, and two patients (6.7%) with FOUR. This is noteworthy, as GCS does not have visual pursuit and fixation criteria, and FOUR doesn't assess visual fixation. 23 These findings underscore the scale's heightened sensitivity in detecting signs of consciousness, confirming the American 6 and European 7 guidelines that endorse CRS-R as the gold standard for DoC assessment.

Potential limitations of this study include the possible inexperience of the professionals involved. Nevertheless, diagnostic changes were noted in just four instances, underscoring the overall reliability of the tool. Another limitation was the inclusion of some patients who presented with pain, seizures, and sleep problems, since treatment with drugs for these conditions can influence the results as factors such as age, number of days postinjury, and time of the day when the scale was completed can influence the result.

In Brazil, accurately identifying consciousness in patients with severe brain injuries, especially those who cannot communicate, remains a formidable challenge, leading to frequent misdiagnoses. Many patients do not receive the requisite rehabilitation, adding to the severity of the problem.

Patients with DoC need specific rehabilitation and therapeutic strategies. By understanding the clinical and epidemiological profiles and insights into neurological findings and prevalent symptoms, professionals can more effectively prevent complications and tailor interventions, thereby elevating the quality of life for patients and their families. 24 25 There is an urgent need to advocate for research on therapeutic interventions, symptom management, and preventive measures for this demographic. 26 27 28 The task of spreading awareness about the correct diagnosis of DoC is equally paramount, given the profound repercussions a misdiagnosis can have on patients and their families.

A secondary yet crucial purpose of this study is to emphasize the catastrophic consequences of misdiagnosis and advise Brazilian health professionals to commit to accurate and timely diagnoses.

In conclusion, when administered by trained professionals, the Brazilian Portuguese version of CRS-R is a reliable tool. It demonstrates substantial to almost perfect interrater agreement for both the total score and its subscales. Additionally, it showcases high concurrent validity when compared with two other standardized behavioral scales: GCS and FOUR. Notably, CRS-R is not limited to physicians, and can be employed by various healthcare professionals, reinforcing the tool's versatility. Training programs focusing on CRS-R are essential to improve the assessment and rehabilitation of patients with DoC in Brazil.
