# Supplementary material for: Validation to Brazilian Portuguese of the coma recovery scale-revised
Source: Arq Neuropsiquiatr. 2024 Nov 20;82(11):s00441791657. doi: 10.1055/s-0044-1791657 (PMC11578665; doi:10.1055/s-0044-1791657)
Supplement: Supplementary file 1 — Supplementary Material [file 10-1055-s-0044-1791657-s230315.pdf]

Supplementary Material S1 Escala de Recuperaç ão do Coma - Revisada 2004

Escala de Recuperação do Coma - Revisada 2004<br>Formulário de Registro<br>*Este formulário deve ser usado em associação com ERC-R Administração e Guia de Pontuação que fornece instruções para a aplicação padronizada da escala.*										
**Paciente:**	**Diagnóstico:**									
**Data de início:**	**Data de Admissão:**									
**Data**										
**Avaliação**	**1**		**2**		**3**		**4**		**5**	
**Escala de função auditiva**	**#**	**CCT**	**#**	**CCT**	**#**	**CCT**	**#**	**CCT**	**#**	**CCT**
4 - Movimento consistente ao comando ■										
3 - Movimento reproduzível ao comando■										
2–Localização do som										
1 - Susto (startle) ao som										
0 - Nenhuma resposta										
**Escala de função visual**	**#**	**CCT**	**#**	**CCT**	**#**	**CCT**	**#**	**CCT**	**#**	**CCT**
5–Reconhecimento de objetos■										
4 - Localização de objetos: tenta alcançar*										
3 - Perseguição de objetos visualmente*										
2–Fixação do olhar em objetos*										
1 - Susto (startle) ao estímulo visual										
0–Nenhuma resposta										
**Escala de função motora**	**#**	**CCT**	**#**	**CCT**	**#**	**CCT**	**#**	**CCT**	**#**	**CCT**
6 - Uso funcional do objeto†										
5 - Resposta motora automática*										
4–Manipulação do objeto*										
3–Localização do estímulo doloroso*										
2–Movimento de retirada em flexão										
1 - Posturas anormais										
0 - Nenhuma resposta										
**Escala de função verbal/oromotora**	**#**	**CCT**	**#**	**CCT**	**#**	**CCT**	**#**	**CCT**	**#**	**CCT**
3 - Verbalização de forma inteligível ■										
2–Movimentação da cavidade oral e vocalização										
1–Movimentação da cavidade oral de forma reflexa										
0 - Nenhuma resposta										
**Escala de comunicação**	**#**	**CCT**	**#**	**CCT**	**#**	**CCT**	**#**	**CCT**	**#**	**CCT**
2 - Comunicação de forma funcional e precisa †										
1 - Comunicação não funcional: intencional■										
0 - Nenhuma resposta										
**Escala do nível de consciência / despertar**	**#**	**CCT**	**#**	**CCT**	**#**	**CCT**	**#**	**CCT**	**#**	**CCT**

(*Continued*)

(*Continued*)

**Escala de Recuperação do Coma - Revisada 2004**<br>**Formulário de Registro**<br>***Este formulário deve ser usado em associação com ERC-R Administração e Guia de Pontuação que fornece instruções para a aplicação padronizada da escala.***										
3. Atenção										
2. Abertura ocular sem estímulo										
1. Abertura ocular com estímulo										
0. Nenhuma resposta										
**TOTAL**										

* Estado Mínimo de Consciência Minus (EMC –).
■ Estado Mínimo de Consciência Plus (EMC +).
† Emergência do Estado Mínimo de Consciência (eEMC).
